# Olive Oil and Nuts in Rheumatoid Arthritis Disease Activity

**DOI:** 10.3390/nu15040963

**Published:** 2023-02-15

**Authors:** Roberta De Vito, Federica Fiori, Monica Ferraroni, Silvia Cavalli, Roberto Caporali, Francesca Ingegnoli, Maria Parpinel, Valeria Edefonti

**Affiliations:** 1 Department of Biostatistics and Data Science Initiative, Brown University, 121 South Main Street and 164 Angell Street, Providence, RI 02912, USA; 2 Department of Medicine—DAME, University of Udine, Via Colugna 50, 33100 Udine, Italy; 3 Branch of Medical Statistics, Biometry, and Epidemiology “G.A. Maccacaro”, Department of Clinical Sciences and Community Health, Università degli Studi di Milano, Via Celoria 22, 20133 Milan, Italy; 4 Fondazione IRCCS Ca’ Granda Ospedale Maggiore Policlinico, Via Sforza 35, 20122 Milan, Italy; 5 Clinical Rheumatology Unit, ASST Gaetano Pini, Department of Clinical Sciences and Community Health, Research Center for Adult and Pediatric Rheumatic Diseases, Università degli Studi di Milano, 20122 Milan, Italy

**Keywords:** cross-sectional study, DAS28, dietary habits, disease activity, food groups, olive oil, nuts, rheumatoid arthritis, SDAI

## Abstract

Few observational studies investigated the relationship between single food groups and disease activity in rheumatoid arthritis (RA). Within a recent Italian cross-sectional study (365 patients, median age: 58.46 years, 78.63% females), we focused on two food groups, olive oil and nuts, representing vegetable sources of fatty acids. Disease activity was measured with Disease Activity Score on 28 joints based on C-reactive protein (DAS28-CRP) and the Simplified Disease Activity Index (SDAI). Robust linear and logistic regression models included tertile-based consumption categories of each food group and several confounders. Stratified analyses were performed by disease severity or duration. Higher consumption of both food groups exerted a favorable effect on disease activity, significant only for olive oil (Beta: −0.33, *p*-value: 0.03) in the linear regression on the overall sample. This favorable effect was stronger in the more severe or long-standing forms of RA (*p*-value for heterogeneity <0.05, especially for disease severity). Significant ORs were as low as ~0.30 for both food groups, strata (i.e., more severe and long-standing RA), and disease activity measures. Mean DAS28-CRP significantly decreased by ~0.70 for olive oil and ~0.55 for nuts in the two strata; mean SDAI significantly decreased by 3.30 or more for olive oil in the two strata. Globally, the beta coefficients doubled, and the ORs halved (in absolute values) for both food groups, reaching significance in 12 of the 16 available models fitted to the more severe or long-standing RA strata. More compromised forms of RA may benefit from increasing consumption of olive oil, olives, and nuts.

## 1. Introduction

Rheumatoid arthritis (RA) is a chronic, autoimmune arthropathy and systemic rheumatic disease, affecting 1% of the general population [1]. The main target of the disease is the synovial membrane of diarthrodial joints, which undergoes inflammation and subsequent hypertrophy. If untreated, progressive damage of the whole joint tissues (synovial membrane, cartilage, and bone) occurs, leading to permanent deformity and disability [1]. Given its high prevalence, RA management should represent a fundamental issue for every medical practitioner.

RA treatment is based on the concept of “treat to target”, implying disease activity scores (e.g., Simplified Disease Activity Index (SDAI), Disease Activity Score on 28 joints (DAS28)) used to reach the goal of remission or a lower disease activity state (LDA). This management strategy limits disease activity, damage, and radiographic progression [2]. While significant steps have been recently made in medical therapy, mainly regarding modern biological and targeted synthetic Disease Modifying Anti-Rheumatic Drugs (DMARDs), a non-negligible portion of patients still does not reach the LDA. Despite several courses of therapies, some of them remain with symptoms and are therefore indicated as “difficult to treat” according to the European Alliance of Associations for Rheumatology (EULAR) definition [2,3].

Increasing interest is rising in lifestyle behaviors, including dietary habits, as complementary support to drug-based treatment of RA. Recent EULAR recommendations highlight the importance of intervening on modifiable risk factors [4]. Among them, smoking, physical inactivity, and an unhealthy or unbalanced diet may have a major role in RA disease activity [5]. Modifiable risk factors are critical if one considers that RA patients have a higher risk of cardiovascular disease, compared to the general population, with cardiovascular disease likely ending up in premature death for these patients [6]. In this setting, RA patients often ask for dietary advice from their professional medical team: this can be considered an unmet need [4,7].

The scientific literature has suggested that following the Mediterranean diet benefits many rheumatologic ongoing conditions, including RA [8]. Indeed, Mediterranean diet can help control well-known RA comorbidities (hypertension, hyperlipidemia, diabetes, and coronary artery disease) and cardiovascular disease risk. Moreover, the Mediterranean diet favors better outcomes of the underlying inflammatory disease [9].

The anti-inflammatory role of Mediterranean diet has been hypothesized to be mainly related to the provided fatty acid profile—monounsaturated fatty acids (MUFAs) and polyunsaturated fatty acids (PUFAs)—the ratio of vitamin E to PUFAs, and phenolic compounds. These profiles share anti-oxidant properties and are mainly contained in extra virgin olive oil and dried fruit rich in omega-3 fatty acids [10]. In vitro and in vivo models have been widely used to investigate these anti-inflammatory compounds’ cellular and biological basis, showing potential reduction in pro-inflammatory cytokines either at the blood level or in the joints. Moreover, these compounds likely hamper pro-inflammatory enzymes (e.g., COX2 and metalloproteases) expression and leukocyte infiltration in the synovial membrane [11]. Finally, dietary fibers from fruit and vegetables can beneficially affect the gut microbiota, thus leading to a further decrease in systemic inflammation [12]. However, evidence from studies in humans considering dietary habits or interventions in RA disease activity [4,8,13,14,15,16] has confirmed only in part results from in vivo and in vitro studies, likely due to severe methodological flaws and heterogeneity of targeted populations.

In this cross-sectional analysis of RA patients from Italy, where extra virgin olive oil is traditionally used [17], we aim to assess if consumption of two food groups, one including olive oil and olives, and the other considering six types of nuts, might improve the control of RA disease activity, through the related higher intake of vegetable-source fatty acids.

## 2. Materials and Methods

### 2.1. Design and Participants

The study population was derived from an observational, cross-sectional, single-center study carried out at the in- and out-patient rheumatology clinic, Gaetano Pini Hospital, Milan, Italy. The recruitment was conducted from January 2018 to December 2019. The study was authorized by the local ethics committee (granted ethical approval: 751_2017, Comitato Etico Milano Area 2) of the Milan area in charge of evaluating protocols from the Pini Hospital. All recruited patients were between 18 and 65 years, had a minimal disease duration of 3 months, and conformed to the 1987 American College of Rheumatology (ACR) [18] and/or the 2010 ACR/European League Against Rheumatism classification criteria [19] for RA.

Specific characteristics of study participants and results on the relation between *a priori* (i.e., Mediterranean diet) and *a posteriori* dietary patterns (derived from the application of factor analysis) were introduced in two recent papers [20,21] that consider the same target population.

### 2.2. Data Collection

Briefly, information from participants enrolled in the current study was obtained from the first routine doctor visit within the recruitment window by centrally trained interviewers. A comprehensive evaluation allowed to collect details on sociodemographic characteristics, anthropometric factors, cigarette smoking and alcohol drinking, and a personal medical history of selected comorbidities. The RA disease activity (measured with both DAS28 with C-reactive protein (CRP) (named DAS28-CRP from here after) and SDAI indexes), ongoing RA treatment, a score of patient’s general health (0 (worst imaginable health state)—100 (best imaginable health state)), and a score for physician’s global assessment (0 (best disease control)—10 (worst disease control)) were evaluated during the same reference visit.

A 110-item food frequency questionnaire (FFQ) was also administered during the visit, inquiring about dietary habits in the previous six months. The FFQ covered the following sections: (1) meat and fish products; (2) sweets, nuts, and snacks; (3) oils and seasonings; (4) vegetables; (5) fruit; (6) drinks; (7) cereals and starchy foods; and (8) milk and dairy products. Study subjects reported their intakes of foods and beverages, answering closed-form frequency questions. For most questions frequency categories ranged from “never” to “4–5 times per day”. Each FFQ item provided also a medium serving size in grams (e.g., mushrooms, 150 g) or in natural units (e.g., tea, one cup). Consumption of seasonal foods was indicated in the FFQ to take into account food supply in season; the average food consumption was then properly adjusted for seasonality [22]. Reproducibility and relative validity assessment of the FFQ provided reassuring results, as conducted in an Italian population from Sicily region [22]. Intakes of total energy and selected macro- and micro-nutrients were calculated for each participant by converting FFQ item consumption frequencies (per day) through the food composition tables [23] produced by the Italian Research Center for Foods and Nutrition [24] and the US Department of Agriculture (USDA) National Nutrient Database for Standard Reference, version 2011 [25].

### 2.3. Selection of Participants

We carefully checked FFQ information provided by the 39 subjects presenting at baseline with extreme (i.e., <5th or >95th percentile) total energy intake. Among them, only one participant showed an unreliable FFQ and was excluded. Our analyses were therefore based on a total of 365 subjects. Additional details are provided elsewhere [21].

### 2.4. Specification of Variables: Exposures of Interest

The current analysis considered the effect on RA disease activity of the following food groups (intakes per day): (1) olive oil and olives (hereafter referred to as “olive oil”), which included olive oil, black olives, and green olives, and (2) nuts, which included chestnuts, peanuts, pistachios, walnuts, almonds, and hazelnuts. While black and green olives were reported in the standard 9 frequency categories previously described (i.e., frequencies ranging from “never” to “4–5 times per day”), olive oil consumption was captured with the following 4 frequency categories: “never”, “less than 1 time per day”, “1 time per day”, or “2 or more times per day”, with the reference portion of 1 spoon. For each food item in the nuts food group, the standard 9 frequency categories of consumption were used, with standard portion sizes equal to 6 pieces for chestnuts, 5 for walnuts, and 10 pieces for the remaining items.

### 2.5. Statistical Analysis

For each food group under consideration, we categorized participants based on the corresponding tertiles of consumption. Multiple regression models included tertile-based categories of each food group as the main independent variable (expressed as the two highest categories of consumption vs. the lowest one, considered as the reference category) and RA disease activity as the dependent variable, expressed as either DAS28-CRP or SDAI.

Disease activity was included in the models as a continuous or a binary variable. In detail, when RA disease activity was a continuous variable, the regression models estimated the effect of each one of the two highest consumption categories on the mean increase/decrease in RA disease activity, compared to the lowest category of consumption. We checked the adherence to the standard ordinary least squares assumptions of our linear regression models. Those assumptions were violated in our data, and thus we adopted the robust MM estimator [26]. When the RA disease activity variable was instead binary (i.e., presence of low, moderate, or high disease activity vs. remission), the logistic regression model allowed to estimate the odds ratios (ORs) of RA disease activity (vs. remission) and the corresponding 95% confidence intervals (CIs) for each of the two highest consumption categories of each food group (vs. the reference one).

In each regression model, we adjusted for the same set of potential confounding variables (for categorical variables, categories shown in Table S1): age, sex, education, body mass index (BMI), cigarette smoking status, alcohol drinking intensity (in drinks/day, where 1 drink was equal to 12 g of ethanol in the Italian population [27]), disease duration, rheumatoid factor (RF), anti-citrullinated protein antibodies (ACPA), presence of any therapy, conventional synthetic (cs)DMARDs, biologic (b)DMARDs, targeted synthetic (ts)DMARDs, steroids, and total energy intake.

For each combination of food group and disease activity measure, we performed stratified analyses by disease severity (RF and ACPA negative, RF and/or ACPA positive) and by disease duration (≤15 years, >15 years). We tested for heterogeneity across strata by using the likelihood ratio test. Two sensitivity analyses were also conducted on (1) subjects having normal blood pressure; (2) subjects having gastro-esophageal reflux or gastritis.

Statistical analyses were carried out using the open-source statistical environment R [28] and its libraries “MASS” [29], “robustbase” [30], and “xlsx” [31].

## 3. Results

### 3.1. Study Population Characteristics: Most Highly Educated Females in Remission or with Low Disease Activity

The socio-demographic characteristics of RA patients included in the study are presented in Table S1. Specifically, patient’s median age was 58.46 (Inter Quartile Range (IQR): 47.81–69.03) years, the majority of whom were females (78.63%) and finished high school and/or university (60.55%). Patient’s median BMI was equal to 23.63 (IQR: 21.00–26.78) kg/m^2^, never smokers were 51.64%, and never drinkers were 29.04%. The median disease activity was equal to 2.21 (IQR: 1.61–3.02) for DAS28-CRP and 6.30 (IQR: 3.01–11.81) for SDAI. Disease duration showed a median of 12.81 (IQR: 8.08–20.72) years, with RF positivity in 53.70% and ACPA positivity in 50.96% of the sample. Ninety-two percent of participants were under pharmacological treatment; 65.8% were under treatment with csDMARDs, 48.8% with bDMARDs, 1.9% with tsDMARDs, and 42.7% with steroids. The median energy intake in the sample was equal to 1975.8 (IQR: 1512.4–2454.2) kcal.

### 3.2. Overall Sample: Lower DAS28-CRP for the Highest Intake of Olive Oil within a General Framework of Favorable but Nonsignificant Effects for Both Food Groups

Table 1 (upper panel) presents the ORs and their corresponding 95% CIs of RA disease activity, and (lower panel) the increase in mean disease activity scores, DAS28-CRP and SDAI in continuous value, according to the highest tertile categories of consumption of the two food groups, olive oil and nuts. The results were derived from logistic and robust regression models including the single food group and the set of confounding factors measured on the overall sample. Higher consumption categories of both food groups generally exerted a favorable effect on disease activity, but in most cases in the absence of statistical significance. The strongest favorable effect was observed for the olive oil group on DAS28-CRP, with a beta coefficient of −0.33 (Standard Error (SE): 0.15), and a *p*-value of 0.03 from Student’s t-test for the highest vs. the lowest consumption category (Table 1, lower panel). In other words, being in the highest tertile category of olive oil consumption provided a significant mean reduction in DAS28-CRP of 0.33, compared to the lowest tertile category. This reduction already accounted for the effect of several confounding factors, including any pharmacological therapy.

### 3.3. Strata by Disease Severity: Lower Disease Activity (DAS28-CRP and SDAI) in the Highest Tertile Category of Olive Oil and Nuts Consumption

Table 2 shows results from the stratified analysis on the more severe (i.e., RF and/or ACPA positive subjects, left panel) and the less severe (i.e., RF and ACPA negative subjects, right panel) RA variants for both olive oil and nuts food groups. The beneficial effect of higher consumption of each food group was generally stronger in the more severe form of RA, independently of the outcome measure considered, with heterogeneity across strata significantly detected in four out of the eight available models.

For the olive oil group, the OR of disease activity was equal to 0.32 (95% CI: 0.13–0.81) for DAS28-CRP and to 0.26 (95% CI: 0.10–0.68) for SDAI (*p*-values for heterogeneity equal to 0.09 and 0.01, respectively). This means that, for both disease activity measures, the odds of having an active disease was significantly lower in the highest tertile category of olive oil consumption, compared to the lowest one. Similarly, the beta coefficient was equal to −0.71 (SE: 0.20) for the DAS28-CRP outcome and to −3.32 (SE: 1.16) for the SDAI outcome, both with a *p*-value ≤ 0.01. In other words, being in the third tertile category of consumption was associated with a mean DAS28-CRP reduced by 0.71 and a mean SDAI reduced by 3.32 for olive oil. The *p*-values for heterogeneity across strata were equal to 0.15 and <0.0001 for the two outcomes, respectively.

In the more severe RA stratum (Table 2, left panel), increasing consumption of nuts exerted a significant protection on DAS28-CRP and SDAI in logistic regression models. Specifically, the OR of disease activity was equal to 0.27 (95% CI: 0.11–0.64) for DAS28-CRP and to 0.36 (95% CI: 0.14–0.95) for SDAI; the corresponding *p*-values for heterogeneity across strata were equal to 0.07 and 0.04, respectively. This means that, for both disease activity measures, the odds of having an active disease was significantly lower in the highest tertile category of nuts consumption, compared to the lowest one. Similar but weaker results were obtained from robust regression models: the beta coefficient was −0.53 (SE: 0.20, *p*-value = 0.01) for DAS28-CRP and −1.61 (SE: 1.12, *p*-value = 0.15) for SDAI in the more severe RA variant, with *p*-values for heterogeneity across strata equal to 0.66 and <0.001, respectively. In other words, being in the third tertile category of consumption was associated with a mean DAS28-CRP reduced by 0.53 for nuts.

Compared to the overall analysis, adjusted beta coefficients doubled (or more), and adjusted ORs halved or more (in absolute values) in the more severe RA stratum for both food groups, reaching significance in all models except one.

### 3.4. Strata by Disease Duration: Lower DAS28-CRP in the Highest Tertile Category of Olive Oil and Nuts Consumption

Table 3 shows results from the stratified analysis on longer (i.e., >15 years, left panel) and shorter (i.e., ≤15 years, right panel) RA disease durations for both olive oil and nuts food groups. Results were consistent with those from the stratified analysis on disease severity: the favorable effects of higher consumption categories of the olive oil and nuts food groups were stronger in the long-standing forms of RA (Table 3, left panel). For DAS28-CRP, the OR of RA disease activity was 0.29 (95% CI: 0.10–0.87) for the olive oil food group and 0.30 (95% CI: 0.10–0.88) for the nuts food group; this means that the odds of having an active disease (according to DAS28-CRP) was significantly lower in the highest tertile categories of consumption for both olive oil and nuts, compared to the lowest tertile categories. The beta coefficient of disease activity for DAS28-CRP was equal to −0.71 (SE: 0.26) for the olive oil food group and to −0.55 (SE: 0.25) for the nuts food group, both with a *p*-value < 0.05; in other words, being in the third tertile category of consumption was associated with a mean DAS28-CRP reduced by 0.71 for olive oil and by 0.55 for nuts. However, differently from the disease severity stratum, the *p*-values of heterogeneity across strata exceeded 0.05 in all the previous models.

Compared to the more severe RA, results were also weaker for the SDAI activity measure in the long-standing form of RA. The beta coefficient for the olive oil food group was equal to −3.62 (SE: 1.62, *p*-value < 0.05) (i.e., a mean SDAI reduced by 3.62 in the third tertile category) and the corresponding *p*-value for heterogeneity across strata <0.0001, but results from the logistic regressions were nonsignificant. The nuts food group was not associated with SDAI either as a binary or a continuous variable.

Compared to the overall analysis, adjusted beta coefficients doubled (or more), and adjusted ORs halved (in absolute values) in the long-standing RA stratum for both food groups, reaching significance in four additional models.

### 3.5. Sensitivity Analyses: Results in Line with the Main Analysis

In the sensitivity analyses, we focused on patients who either had normal blood pressure or did not report gastro-esophageal reflux or gastritis. The corresponding point estimates were in line with those from the main analysis; the main difference is that the OR for the olive oil group in the DAS28-CRP became significant (OR: 0.44, 95% CI: 0.20–0.95) for subjects reporting no gastro-esophageal reflux (vs. OR: 0.60, 95% CI: 0.31–1.17 in the complete case analysis). Moreover, the CIs in the stratified analyses were wider in most cases, and sometimes this led to CIs including 1 for the ORs or 0 for the beta coefficients. For example, for DAS28-CRP, normal blood pressure subjects with disease duration >15 years presented an OR of disease activity for the nuts food group of 0.42 (95% CI: 0.12–1.42) vs. 0.30 (95% CI: 0.10–0.86) in the complete case analysis. When we considered SDAI and the nuts food group, normal blood pressure subjects with the more severe RA form presented an OR of 0.39 (95% CI: 0.14–1.03) vs. 0.36 (95% CI: 0.14–0.95), losing the significance.

## 4. Discussion

In the present study on Italian RA patients, higher consumption of the olive oil and nuts food groups generally exerted a favorable effect on disease activity, but, in most cases, in the absence of statistical significance. The favorable effect was, however, stronger for those patients with a more severe RA form or a long-standing RA activity, in the presence of significant heterogeneity across strata, especially for disease severity. In these strata, the betoefficients doubled (or more), and the ORs halved or more (in absolute values) for both food groups, reaching significance in 12 of the 16 available models.

The increasing awareness of an unmet need for nutritional counselling in RA management has recently suggested increasing evidence collection on the role of dietary habits or dietary interventions on RA disease activity [4,8,13,14,15,16]. It might also be the time for a paradigm shift from short-term, likely temporary, effects of dietary interventions in trials on selected RA populations to long-term, likely modest but persistent, effects of usual dietary habits in observational studies on free-living RA patients [4,32]. A negligible “real-life” dietary-based benefit over time may be more critical in the long period than running after benefits from short-term dietary-based interventions. From a patient-centric and a public health point of view, it would be useful to provide RA patients with simple dietary recommendations targeting inexpensive, easily accessible foods which do not require preparation. This is especially true for patients experiencing more severe forms of RA or who suffer for a longer time. They are, indeed, more likely to struggle with managing daily living activities such as cooking. Among possible foods associated with RA activity in previous observational studies, nonfried fish, rich in omega-3 fatty acids [32], and some components of the Mediterranean diet, especially those targeting oleic acid intake [33], was found to deserve further investigation. We, therefore, hypothesized to assess if nuts, olives, and olive oil in general, as easily accessible foods providing vegetable-source fatty acids, might be related to RA disease activity.

Several studies have previously assessed the role of olive oil, olives, and nuts, alone or as components of the Mediterranean diet, in RA disease activity [20,33,34,35,36,37,38,39,40,41,42,43,44,45,46,47,48]. Except for one Iranian trial dealing with the efficacy of topical olive oil in controlling pain through skin absorption [42], the remaining trials considered a supplementary herbal formulation of olive oil, fig and olive fruits, together with routine DMARDs regimen (vs. routine DMARDs regimen alone) [43], or a combination of fish oil omega-3 fatty acids (3 grs/day) and 9.6 mL of olive oil (vs. fish oil omega-3 fatty acid (3 grs/day) or a soy oil placebo), in addition to the usual medication [41]. Indirect information on the potentially beneficial effects of olive oil may be obtained from trials investigating the effect of fish oil on RA disease activity [34,35,44,45,46,47,48]. In the absence of an ideal placebo for inflammatory disease studies, these studies classically used olive oil as a placebo, based on the idea that MUFAs could be regarded as neutral fatty acids [41]. These results overall suggest that olive oil may provide significant improvements in disease activity according to traditional clinical and laboratory parameters and patient’s satisfaction in activities of daily living, although fish oil omega-3 fatty acids have produced superior results compared to it [8].

To our knowledge, no trials in humans have targeted nuts as foods rich in omega-3 fatty acids. Evidence may be still indirectly obtained from trials proposing a portfolio diet of anti-inflammatory foods (i.e., a high intake of fatty fish, whole grains, fruits, nuts, berries, and canola—but not olive—oil) [36,38] or a Mediterranean-like dietary pattern, where olive oil is traditionally consumed too [39,40]. In a cross-over trial, no significant difference in DAS28 was found between the proposed portfolio diet and a control diet similar to the general dietary intake in Sweden, although it was significantly lower after the intervention than after the control period in the participants who completed both periods [36]. Evidence was similarly weak for the Mediterranean-type diet and RA activity outcomes, including DAS28 [39,40]: favorable effects in the Mediterranean diet arm were either modest and not significant for DAS28 [39] or, when present, hard to generalize to different populations, as the Mediterranean diet was adapted to the Swedish population [40].

Finally, few observational studies [20,33,37] considered the relationship between olive oil and/or the Mediterranean diet and disease activity. In two of them—one cross-sectional study on a subset of this database [20] and a case-control study from Japan [33]—the total Mediterranean diet score was not significantly related to composite measures of disease activity. However, when separately analyzed, two single components of DAS28 and SDAI (patient’s general health and global assessment) were significantly related to the total Mediterranean diet score, with higher adherence associated with a better general health index and disease activity [20]. Similarly, single components of the Mediterranean diet might exert modest favorable effects on DAS28: after the model selection was performed on each component in multiple models already adjusted for age, sex, disease duration, and medication, MUFA intake (higher-than vs. lower-than median intake, with median calculated on the control subjects) was the stronger predictor of DAS28 [33]; in logistic regression, the OR of RA remission for higher-than-median MUFA intake was 1.97 (95% CI: 0.98–3.98), close to reaching significance. Since 90% of Japanese daily MUFA intake is likely to be oleic acid and the main sources of oleic acid in Japan are plants, olive oil, avocados, and animal fats (such as lard), this result [33] is in line with our modest (nonsignificant) favorable effect of olive oil and nuts food groups in the overall sample.

Olive oil composition and its potential health effects have been deeply investigated in the past decades. Several studies have found a beneficial effect of olive oil consumption on markers of inflammation, indicating that olive oil is a key ingredient in the overall Mediterranean diet [49]. Its high oleic acid content has been related to decreased adhesion molecules’ expression in peripheral blood mononuclear cells in humans [50]. In addition, olive oil contains polyphenolic bioactive compounds, such as oleuropein, ligstroside, and oleocanthal, and their derivatives phenolic alcohols, which may exert anti-inflammatory effects too [11]. In RA, diets rich in olive oil may exert beneficial effects through its polyphenols: in animal models, they have been shown to modulate different signaling pathways and the related transcription factors, thus reducing inflammatory cytokines expression [51,52,53]. The high antioxidant activity of tyrosol, hydroxytyrosol [54,55,56], and oleuropein [57,58] from olive oil has been shown in several studies. Even if it is still unclear whether olive oil’s effects are due to its fatty acid composition, its bioactive compounds, or both, these mechanisms may explain the decreasing trend in RA disease activity when olive oil was used alone or in combination with omega-3 fatty acids [34,35,41,43,44,45,46,47,48].

Nuts composition is characterized by variable amounts of omega-6 and omega-3 essential fatty acids. The alpha-linolenic (omega-3) fatty acid, contained mainly in walnuts, has been inversely associated with inflammatory markers [59]. In our sample, consumers of walnuts accounted for 49.2% of total nuts consumption; this percentage is higher than the Italian domestic retail data obtained in 2021 [60]. This prevalence of walnuts over the other nuts suggests that walnuts are the major contributor of alpha-linolenic fatty acid intake. Together with linoleic fatty acid and vitamin E, alpha-linolenic fatty acid characterized the “Vegetable unsaturated fatty acids” dietary pattern, found to be inversely related to DAS28-CRP in a previous analysis based on our overall sample, and in the more severe or long-standing RA strata [21]. In the overall diet, the omega-6/omega-3 ratio (ideally 4:1) modulates eicosanoid activity in inflammatory and immune responses [61]. This is witnessed by inflammatory markers improvement in the US population from the Nurses’ Health Study, when three portions of red meat/processed meat/eggs/refined grains were replaced with three servings of nuts per week [59]. The beneficial effect of nuts may also be explained by their flavonoid content. Such compounds may activate antioxidant pathways that render an anti-inflammatory effect and eventually reduce pain and inflammation in joints [62].

The current study has some strengths. In the absence of strong previous evidence on the association between food groups and RA activity, our analysis plan was meant to improve as much as possible results validity. Dietary information was assessed with an FFQ whose reproducibility and validity have been previously evaluated with reassuring results [22]. We considered two well-known composite measures for the assessment of RA disease activity [20]. We also proposed two parallel analyses with logistic and robust linear regression models. It was reasonable to dichotomize disease activity as patients on moderate or high disease activity were just 21 and 28% of the total frequency for each outcome, vs. 62 and 30% patients in remission, respectively. However, logistic regression may provide a raw picture of the association (i.e., remission vs. active disease), whereas beta coefficients from robust linear regression can be easily compared with available thresholds of minimal clinically important improvements in DAS28 and SDAI from clinical trials [63]. We also conducted two sensitivity analyses to reduce the possible effects of additional comorbidities present in the study sample. The consistency of our findings across the different analyses performed is reassuring.

Among study limitations, we first acknowledge our cross-sectional study design, which does not allow us to draw firm conclusions on the relationship between dietary habits and RA disease activity. On the other hand, in retrospective study designs on RA disease progression, affected subjects would be even more likely to have biased recall of dietary exposures than in other diseases. Prospective study designs in the era of effective DMARDs may require large sample sizes to detect small differences in disease activity related to dietary habits, and to adjust for the several confounding factors, including drugs, that influence disease activity [13]. In addition, identifying the appropriate time window between dietary exposure and disease activity change is critical, which make it difficult to properly design studies on diet and RA disease activity. “Chronic” dietary exposures—for example, adherence to particular dietary patterns or frequent consumption of certain foods—may impact a patient’s general state of disease activity over many months [13] and thus exceed the reference period of the FFQ used for dietary assessment. Similarly, “chronic” dietary exposures may have been originally responsible for RA onset and, if persisting over time, they are still responsible for RA progression over the investigated time frame. In crossectional studies on RA patients, it is, therefore, more difficult to distinguish between dietary patterns potentially related to incidence and those related to the progression of the disease. Finally, patients may experience fluctuations in symptoms over small periods (e.g., 3 months) and routinely adapt their dietary habits—increasing the consumption of apparently healthy foods and reducing that of detrimental ones—to compensate for varying symptoms. The assessment of “usual” dietary intake typical of FFQs is challenging, even when the reference period is small, as in our study design.

In conclusion, when looking for easily accessible, unexpensive food groups that potentially reduce RA activity, patients might consider using/increasing the consumption of (extra virgin) olive oil, olives, and nuts, especially when RA is severe or affects patients for a long time. These results need confirmation based on additional well-designed cohort studies referring to validated dietary assessment tools and objective measures of disease activity, administered to a large population, to find the expected small dietary effects and to adjust for the large set of confounding factors typical of studies on RA disease activity. Future studies should also investigate RA comorbidities-related outcomes [64].

## Figures and Tables

**Table 1:** Odds ratio of rheumatoid arthritis disease activity and corresponding 95% confidence intervals (CI) (upper panel) and increment in the mean DAS28-CRP and SDAI in continuous (lower panel), according to the highest tertile-based categories of consumption of the two food groups, olive oil and nuts. Overall analysis. Italy 2018–2019.

Overall Analysis				
**Logistic Regression**				
**Food Groups**	**Tertile Categories**	**OR**	**95% CI**	
**DAS28-CRP**				
Olive oil	I [0, 2.06]	1 ^1^		
	II (2.06, 3.06]	0.93	0.52	1.64
	III (3.06, 6.99]	0.60	0.31	1.17
Nuts	I [0, 0.1]	1^1^		
	II (0.1, 0.42]	0.71	0.38	1.30
	III (0.42, 13.5]	0.57	0.31	1.06
**SDAI**				
Olive oil	I [0, 2.06]	1 ^1^		
	II (2.06, 3.06]	0.99	0.51	1.91
	III (3.06, 6.99]	0.69	0.34	1.40
Nuts	I [0, 0.1]	1 ^1^		
	II (0.1, 0.42]	1.24	0.63	2.43
	III (0.42, 13.5]	1.05	0.54	2.05
**Robust Linear Regression**				
**Food Groups**	**Tertile Categories**	**Beta**	**SE**	***p*-Value**
**DAS28-CRP**				
Olive oil	II (2.06, 3.06]	−0.14	0.13	0.30
	III (3.06, 6.99]	−0.33	0.15	0.03
Nuts	II (0.1, 0.42]	−0.07	0.14	0.64
	III (0.42, 13.5]	−0.20	0.14	0.15
**SDAI**				
Olive oil	II (2.06, 3.06]	−0.53	0.77	0.50
	III (3.06, 6.99]	−1.27	0.87	0.15
Nuts	II (0.1, 0.42]	0.25	0.81	0.76
	III (0.42, 13.5]	−0.26	0.81	0.75

^1^ Reference category.

**Table 2:** Odds ratio of rheumatoid arthritis disease activity and corresponding 95% confidence intervals (CI) (upper panel) and increment in the mean DAS28-CRP and SDAI in continuous (lower panel), according to the highest tertile-based categories of consumption of the two food groups, olive oil and nuts. Stratified analysis by disease severity (RF and ACPA negative, RF and/or ACPA positive). Italy 2018–2019.

		RF and/or ACPA Positive			RF and ACPA Negative			
**Logistic Regression**								
**Food Groups**	**Tertile Categories**	**OR**	**95% CI**		**OR**	**95% CI**		**p_hetero_^1^**
**DAS28-CRP**								
Olive oil	I [0, 2.06]	1 ^2^			1 ^2^			
	II (2.06, 3.06]	0.67	0.31	1.47	2.48	0.74	8.34	
	III (3.06, 6.99]	0.32	0.13	0.81	0.98	0.25	3.90	0.09
Nuts	I [0, 0.1]	1^2^			1^2^			
	II (0.1, 0.42]	0.52	0.23	1.20	0.74	0.25	2.23	
	III (0.42, 13.5]	0.27	0.11	0.64	1.20	0.42	3.47	0.07
**SDAI**								
Olive oil	I [0, 2.06]	1^2^			1^2^			
	II (2.06, 3.06]	0.98	0.37	2.60	1.86	0.54	6.37	
	III (3.06, 6.99]	0.26	0.10	0.68	4.38	0.97	19.72	0.01
Nuts	I [0, 0.1]	1 ^2^			1 ^2^			
	II (0.1, 0.42]	0.48	0.18	1.28	3.72	1.10	12.63	
	III (0.42, 13.5]	0.36	0.14	0.95	2.95	0.87	10.04	0.04
**Robust Linear Regression**								
**Food Groups**	**Tertile Categories**	**Beta**	**SE**	***p*-Value**	**Beta**	**SE**	***p*-Value**	**p_hetero_^1^**
**DAS28-CRP**								
Olive oil	II (2.06, 3.06]	−0.22	0.19	0.24	0.24	0.21	0.25	
	III (3.06, 6.99]	−0.71	0.20	0.00	0.18	0.24	0.45	0.15
Nuts	II (0.1, 0.42]	−0.25	0.20	0.21	0.18	0.20	0.37	
	III (0.42, 13.5]	−0.53	0.20	0.01	0.12	0.19	0.53	0.66
**SDAI**								
Olive oil	II (2.06, 3.06]	−1.65	1.07	0.13	1.62	1.31	0.22	
	III (3.06, 6.99]	−3.32	1.16	0.01	0.01	1.50	0.99	<0.0001
Nuts	II (0.1, 0.42]	0.02	1.11	0.99	0.71	1.25	0.58	
	III (0.42, 13.5]	−1.61	1.12	0.15	0.56	1.22	0.64	<0.0001

^1^*p*-value for heterogeneity of effects estimates across strata. ^2^ Reference category.

**Table 3:** Odds ratio of rheumatoid arthritis disease activity and corresponding 95% confidence intervals (CI) (upper panel) and increment in the mean DAS28-CRP and SDAI in continuous (lower panel), according to the highest tertile-based categories of consumption of the two food groups, olive oil and nuts. Stratified analysis by disease duration (≤15 years, >15 years). Italy 2018–2019.

		Disease Duration > 15 Years			Disease Duration ≤ 15 Years			
**Logistic Regression**								
**Food Groups**	**Tertile Categories**	**OR**	**95% CI**		**OR**	**95% CI**		**p_hetero_^1^**
**DAS28-CRP**								
Olive oil	I [0, 2.06]	1 ^2^			1 ^2^			
	II (2.06, 3.06]	0.56	0.21	1.54	1.34	0.57	3.14	
	III (3.06, 6.99]	0.29	0.10	0.87	0.82	0.29	2.27	0.50
Nuts	I [0, 0.1]	1^2^			1^2^			
	II (0.1, 0.42]	0.59	0.21	1.65	0.63	0.25	1.57	
	III (0.42, 13.5]	0.30	0.10	0.86	0.77	0.29	2.05	0.56
**SDAI**								
Olive oil	I [0, 2.06]	1^2^			1^2^			
	II (2.06, 3.06]	0.47	0.24	0.92	0.99	0.62	1.60	
	III (3.06, 6.99]	0.62	0.18	2.11	0.50	0.20	1.25	0.13
Nuts	I [0, 0.1]	1^2^			1^2^			
	II (0.1, 0.42]	0.90	0.51	1.59	0.97	0.61	1.55	
	III (0.42, 13.5]	0.45	0.14	1.46	0.50	0.20	1.27	0.79
**Robust Linear Regression**								
**Food Groups**	**Tertile Categories**	**Beta**	**SE**	***p*** **-Value**	**Beta**	**SE**	***p*** **-Value**	**p_hetero_^1^**
**DAS28-CRP**								
Olive oil	II (2.06, 3.06]	−0.35	0.24	0.16	−0.04	0.16	0.82	
	III (3.06, 6.99]	−0.71	0.26	0.01	−0.08	0.18	0.65	0.47
Nuts	II (0.1, 0.42]	−0.06	0.25	0.80	−0.16	0.17	0.36	
	III (0.42, 13.5]	−0.55	0.25	0.03	−0.15	0.18	0.39	0.62
**SDAI**								
Olive oil	II (2.06, 3.06]	−2.00	1.49	0.19	0.54	0.93	0.57	
	III (3.06, 6.99]	−3.62	1.62	0.03	−0.10	1.05	0.92	<0.0001
Nuts	II (0.1, 0.42]	1.14	1.53	0.46	−0.62	0.95	0.52	
	III (0.42, 13.5]	−1.50	1.51	0.33	−0.55	1.01	0.59	1

^1^*p*-value for heterogeneity of effects estimates across strata. ^2^ Reference category.

## Data Availability

Data used in this study are not publicly available, but can be obtained upon reasonable request.

## Supplementary Materials

The following supporting information can be downloaded at: https://www.mdpi.com/article/10.3390/nu15040963/s1, Table S1: Distribution of 365 rheumatoid arthritis patients according to selected characteristics. Italy 2018–2019.
