# Supplementary material for: Olive Oil and Nuts in Rheumatoid Arthritis Disease Activity
Source: Nutrients. 2023 Feb 15;15(4):963. doi: 10.3390/nu15040963 (PMC9962234; doi:10.3390/nu15040963)
Supplement: Supplementary file 1 [file nutrients-15-00963-s001.zip › nutrients-2163704-supplementary.pdf]

Table S1. Distribution of 365 rheumatoid arthritis patients according to selected characteristics. Italy 2018-2019.

**Characteristics**	
**Age at baseline**, years, median (IQR)[1]	58.46 (47.81-69.03)
≤55 years, N (%)	155 (42.47)
>55, N (%)	210 (57.53)
**Female**, N (%)	287 (78.63)
**Education**[2]	
Primary school, N (%)	49 (13.42)
Middle school, N (%)	79 (21.64)
High school, N (%)	134 (36.71)
University, N (%)	87 (23.84)
**Body Mass Index**, Kg/m^2, median (IQR)	23.63 (21.00-26.78)
<18.5, N (%)	22 (6.03)
18.5 - 25, N (%)	207 (56.71)
25 - 30, N (%)	94 (25.75)
≥30, N (%)	42 (11.51)
**Cigarette smoking status**[2]	
Never, N (%)	189 (51.78)
Former, N (%)	118 (32.33)
Current, N (%)	55 (15.07)
**Alcohol drinking intensity**	
Never, N (%)	106 (29.04)
<1 drink/day, N (%)	194 (53.15)
1 - 2 drinks/day, N (%)	29 (7.95)
≥2 drinks/day, N (%)	36 (9.86)
**Disease duration**, years, median (IQR)	12.81 (8.08-20.72)
≤5, N (%)	51 (13.97)
5 - 10, N (%)	84 (23.01)
10 - 15, N (%)	76 (20.82)
15 - 25, N (%)	89 (24.38)
>25, N (%)	65 (17.81)
Positivity for **rheumatoid factor**, N (%)	196 (53.70)
Positivity for **anti-citrullinated protein antibodies**, N (%)	186 (50.96)
**DAS28-CRP**, median (IQR)	2.21 (1.61-3.02)
Remission, N (%)	227 (62.19)
Low activity, N (%)	60 (16.44)
Moderate activity, N (%)	64 (17.53)
High activity, N (%)	14 (3.84)
**SDAI**, median (IQR)	6.30 (3.01-11.81)
Remission, N (%)	108 (29.59)
Low activity, N (%)	155 (42.47)
Moderate activity, N (%)	81 (22.19)

High activity, N (%)	21 (5.75)
**Swollen joint count** (0-28), median (IQR)	0 (0-1)
**Tender joint count** (0-28), median (IQR)	0 (0-2)
**C-reactive protein**, mg/dL, median (IQR)	2 (0.6-5.57)
**General Health** (0-100), median (IQR)	70 (60-85)
**Physician's Global Assessment** (0-10), median (IQR)	2 (0-4)
**Comorbidities**	
Chronic renal failure, N (%)	4 (1.1)
Arterial hypertension, N (%)	122 (33.42)
Coronary artery disease, N (%)	19 (5.21)
Diabetes mellitus, N (%)	19 (5.21)
Gastro-esophageal reflux disease, N (%)	70 (19.18)
Inflammatory bowel disease, N (%)	2 (0.55)
Gastritis, N (%)	32 (8.77)
Esophagitis, N (%)	5 (1.37)
**Presence of any therapy**	
No, N (%)	30 (8)
Yes, N (%)	335 (92)
**Conventional Synthetic (cs)DMARDs**[3]	
No, N (%)	118 (34.2)
Yes, N (%)	247 (65.8)
**Biologic (b)DMARDs**[3]	
No, N (%)	187 (51.2)
Yes, N (%)	178 (48.8)
**Targeted Synthetic (ts)DMARDs**[3]	
No, N (%)	358 (98.1)
Yes, N (%)	7 (1.9)
**Steroids**	
No, N (%)	209 (57.3)
Yes, N (%)	156 (42.7)
**Total energy intake at baseline**, kcal, median (IQR)[1]	1975.8 (1512.4-2454.2)

[1] Age was calculated as the difference between date of interview and date of birth; [2]The sum does not add to the total because of a few missing values in education (16 subjects, 4.38%) and cigarette smoking status (3 subjects, 0.82%); [3] Disease Modifying Anti-Rheumatic Drugs.
